# Emerging Potential of Microwave Ablation for Primary Aldosteronism Resulting From Unilateral Aldosterone-producing Adenoma

**DOI:** 10.1210/jcemcr/luad077

**Published:** 2023-07-12

**Authors:** Edouard G Mills, Fausto F Palazzo, Edward Leen, Florian Wernig

**Affiliations:** Section of Investigative Medicine and Endocrinology, Imperial College London, London W12 0NN, UK; Department of Endocrinology, Imperial College Healthcare NHS Trust, London W12 0HS, UK; Department of Endocrine and Thyroid Surgery, Imperial College Healthcare NHS Trust, London W12 0HS, UK; Imaging Department, Imperial College Healthcare NHS Trust, London W12 0HS, UK; Department of Endocrinology, Imperial College Healthcare NHS Trust, London W12 0HS, UK

**Keywords:** primary aldosteronism, Conn’s syndrome, thermal ablation, microwave ablation

## Abstract

Primary aldosteronism (PA) is the most prevalent form of secondary hypertension and is most commonly caused by an adrenal adenoma or bilateral adrenal hyperplasia. Minimally invasive adrenalectomy is the treatment of choice for unilateral disease. Here, we report the case of a 57-year-old man with previous bladder cancer who was referred for evaluation of resistant hypertension and hypokalemia. Diagnostic workup indicated PA with computed tomography imaging revealing a left adrenal adenoma and adrenal venous sampling lateralizing to the left adrenal. He was therefore referred for a left adrenalectomy using a retroperitoneoscopic approach. However, surgery was complicated by significant perinephritis related to previous bladder cancer immunotherapy and, in view of an identifiable adrenal adenoma, a partial adrenalectomy was performed. Despite histology confirming removal of an adrenal adenoma, he remained hypertensive and hypokalemic with persistent PA. He underwent a computed tomography-guided percutaneous thermal (microwave) ablation of the residual adrenal nodule with immediate biochemical reversal of PA. Six years postprocedure, he remains biochemically and clinically cured from PA. This article presents the details of the case and reviews the literature on long-term outcomes for patients undergoing thermal ablation and adrenalectomy, suggesting that thermal ablation may be a viable alternative for selected patients.

## Introduction

Primary aldosteronism (PA) is characterized by autonomous aldosterone secretion from the adrenal glands, resulting in hypertension and in more severe cases hypokalemia. It is most commonly caused by an aldosterone-producing adenoma (APA), unilateral or bilateral adrenal hyperplasia, or, in rare cases, an aldosterone-producing adrenal carcinoma or familial form of aldosteronism. Estimates identify PA in 5% to 15% of hypertensive adults, making it the most common cause of secondary hypertension [1]. Importantly, compared with age- and sex-matched patients with essential hypertension and equivalent blood pressure changes, those with PA are at excess risk of cardiovascular morbidity and mortality [1].

From a therapeutic perspective, subtype differentiation of unilateral from bilateral forms is critical given that for unilateral disease, adrenal surgery offers the possibility of cure, whereas bilateral disease is treated primarily with mineralocorticoid receptor blockade [1]. Here, we present the case of a patient with unilateral PA resulting from an APA who received computed tomography (CT)-guided percutaneous thermal (microwave) ablation after unsuccessful adrenal surgery and is now cured from PA. This highlights that, in selected patients, thermal ablation may serve as an emerging and efficacious alternative to surgery.

## Case Presentation

A 57-year-old Caucasian man was referred to our tertiary endocrinology clinic for evaluation of persistent and resistant hypertension associated with hypokalemia. At referral, despite taking dual antihypertensives at maximal dose (amlodipine 10 mg once daily and doxazosin 8 mg twice daily), his average home blood pressure readings were >160/90 mm Hg. His medical history was significant for asthma and transitional cell carcinoma of the bladder 9 years previously (cured with surgery, chemotherapy, and bacillus Calmette-Guérin immunotherapy).

## Diagnostic Assessment

**Figure 1.**
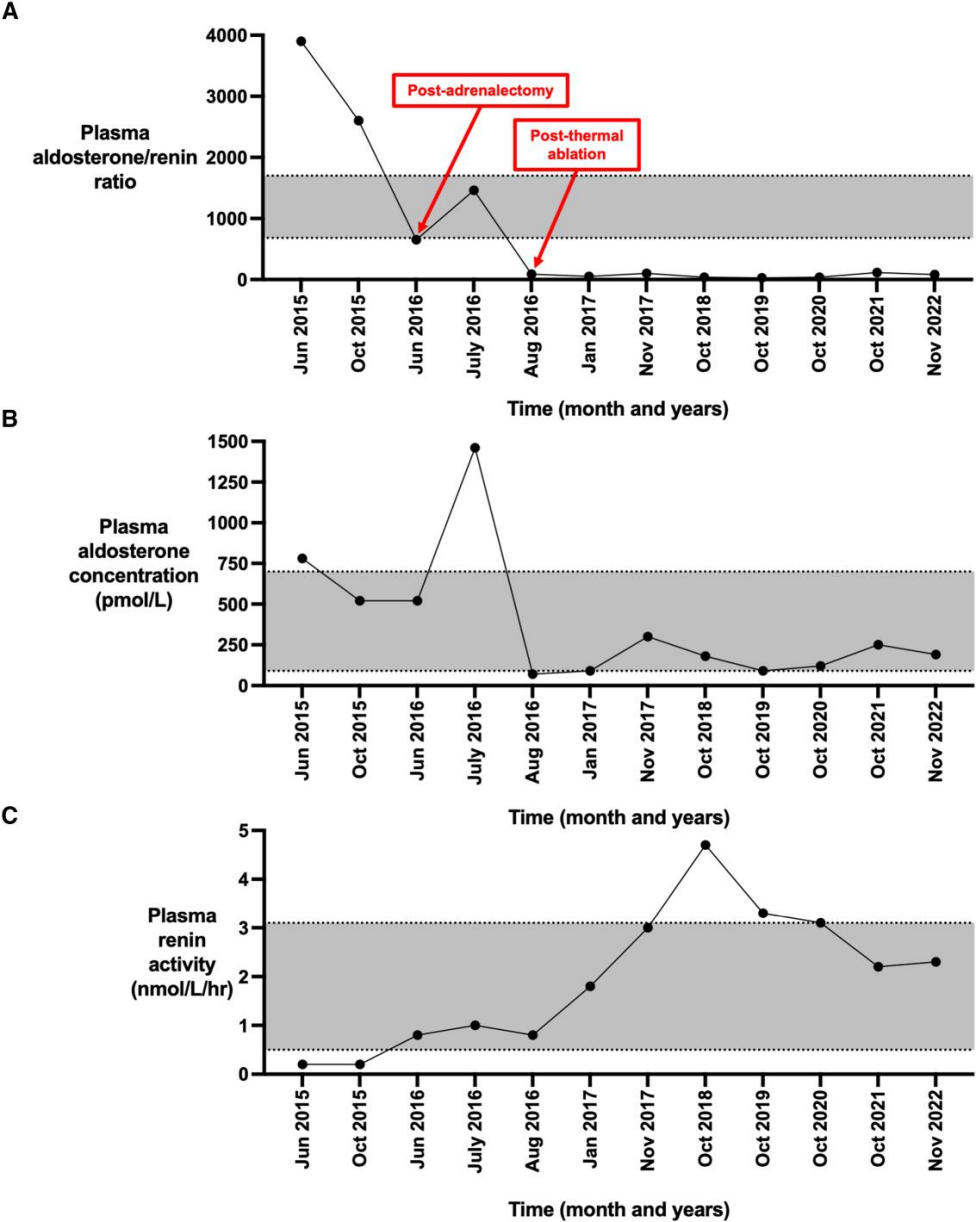
Profiles for plasma aldosterone:renin ratio, plasma aldosterone concentration, and plasma renin activity from June 2015 to November 2022 in relation to the timings of adrenalectomy (June 2016) and thermal ablation (August 2016). (A) Plasma aldosterone:renin ratio (ARR). The upper dotted horizontal line at >1700 indicates an ARR suggestive of PA. The lower dotted horizonal line at <680 indicates an ARR not suggestive of PA. (B) Plasma aldosterone concentration (in pmol/L). The upper dotted horizontal line at 700 pmol/L indicates the upper reference range. The lower horizonal line at 90 pmol/L indicates the lower reference range. (C) Plasma renin activity (in nmol/L/h). The upper dotted horizontal line at 3.1 nmol/L/h indicates the upper reference range. The lower horizonal line at 0.5 nmol/L/h indicates the lower reference range.

Once the patient’s hypokalemia was corrected, a series of laboratory examinations for PA was performed. Taken on doxazosin with a serum potassium level of 3.6 mmol/L (reference range [RR] 3.5-5.0), plasma aldosterone was raised at 780 pmol/L (RR 90-700; equivalent to 28.12 ng/dL) and plasma renin activity (PRA) 0.2 nmol/L/h (RR 0.5-3.1), resulting in a markedly raised aldosterone:renin ratio (ARR) of 3900 (RR <680 PA unlikely, 850-1700 possible PA, >1700 PA likely if PRA <0.3 nmol/L/h) (Fig. 1). A 1-mg overnight dexamethasone suppression test was normal (9 Am cortisol <20 nmol/L, <0.72 µg/dL), thereby excluding potentially accompanying cortisol cosecretion. A seated saline infusion test yielded a 4-hour postinfusion plasma aldosterone value of 240 pmol/L (8.65 ng/dL), thus confirming PA.

**Figure 2.**
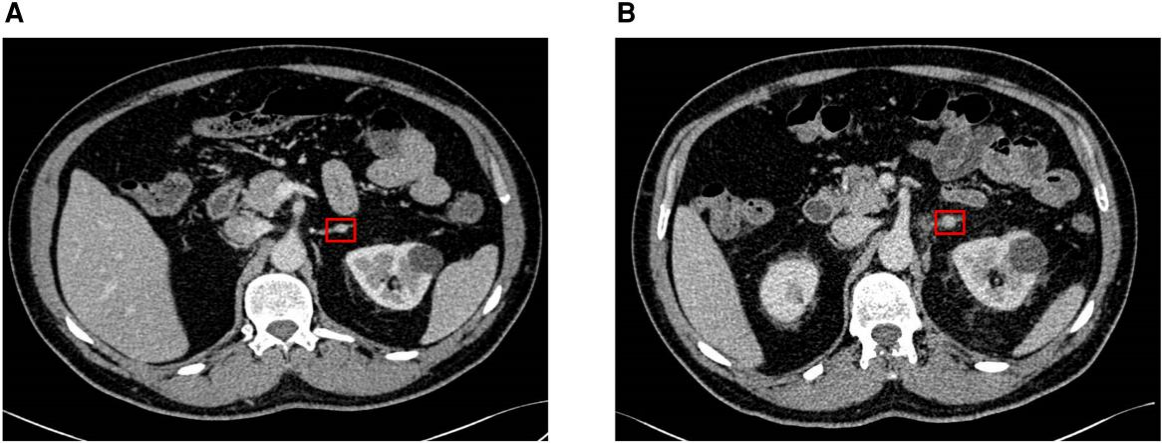
CT adrenal imaging at baseline and in the postoperative period demonstrating a left adrenal adenoma. (A) Baseline imaging demonstrating a 9-mm diameter nodule within the lateral limb of the left adrenal gland. The lesion demonstrated enhancement in keeping with a benign adrenal adenoma (absolute washout 65% and relative washout 55%). Normal configuration of the right adrenal gland. (B) Postoperative imaging demonstrating a partial left adrenalectomy and a residual 6-mm nodule within the lateral limb of the adrenal gland. Boxes in both panels depict left adrenal nodule.

CT adrenal imaging revealed a 9-mm left adrenal lesion (Fig. 2A) with enhancement characteristics consistent with a benign adrenal adenoma (absolute washout 65% and relative washout 55%). Subsequent adrenal venous sampling without tetracosactin stimulation demonstrated lateralization to the left adrenal gland with suppression of right adrenal aldosterone secretion (aldosterone:cortisol ratio in the lower inferior vena cava 1.4, left adrenal vein 2.5, and right adrenal vein 0.2) (Table 1), suggestive of left unilateral disease.

**Table 1.** Adrenal venous sampling confirming unilateral left adrenal disease

	Aldosterone (pmol/L)	Cortisol (nmol/L)	Aldosterone/cortisol
LAV	10260	4109	2.5
RAV	1780	7327	0.2
IVC	270	195	1.4

Table displays results of adrenal venous sampling (without tetracosactin stimulation), during which a catheter is inserted via the femoral vein and the adrenal veins are selectively cannulated under X-ray control. Samples are taken for aldosterone (pmol/L) and cortisol (nmol/L) for measurement of the aldosterone:cortisol ratio from the left adrenal vein (LAV), right adrenal vein (RAV), and lower inferior vena cava (IVC). The cortisol values in each adrenal vein are at least 2 times greater than that in the lower IVC, confirming successful cannulation of the adrenal veins

## Treatment

His case was discussed at a Adrenal Multidisciplinary Team meeting. It was agreed that in view of unilateral PA, he should undergo a minimally invasive adrenalectomy and that he was suitable for a retroperitoneoscopic approach. Intraoperatively, he had significant perinephritis with indurated perirenal fat presumed to be related to previous bacillus Calmette-Guérin immunotherapy. A well-defined adrenal nodule was visible; therefore, a decision to perform a partial adrenalectomy was made.

**Figure 3.**
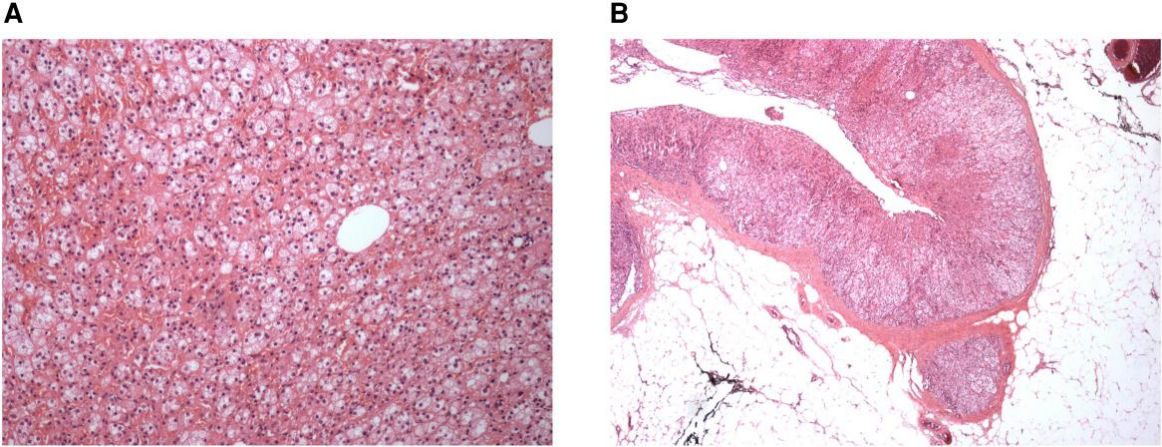
Postoperative adrenal histology. (A) Normal background adrenal architecture. (B) Benign cortical adrenal adenoma.

In the postoperative period, although there was a marginal improvement in the biochemistry and antihypertensive burden, the patient was not cured of PA. At day 14 after surgery, he remained hypokalemic and hypertensive, his blood pressure was 150/90 mm Hg on amlodipine 10 mg (although he was able to discontinue doxazosin) and serum potassium 3.9 mmol/L on oral potassium replacement (1.88 g daily [ie, 48 mmol of K^+^]). Notably, his ARR was lower but remained elevated at 1460 (plasma aldosterone 1460 pmol/L [52.63 ng/dL] and PRA now detectable at 1.0 nmol/L/h) (Fig. 1). Postoperative histology of the resected adrenal tissue showed normal background adrenal architecture and a benign cortical adenoma (Fig. 3). However, postoperative imaging demonstrated a partial left adrenalectomy and a residual 6-mm nodule in the lateral limb of the left adrenal (Fig. 2B), suggesting multiple adenomas (which had not been identified on the preoperative imaging). Given the marginal improvement in his biochemistry and antihypertensive burden in the postoperative period, it is possible that both adenomas were the source of aldosterone hypersecretion.

The treatment options at this point for persistent PA were (1) to redo surgery, (2) long-term medical therapy, or (3) CT-guided percutaneous thermal ablation. Given the technical challenges at his original surgery, reoperation was undesirable, and the option of thermal ablation (using microwave ablation [MWA]) was discussed and agreed on by the Adrenal Multidisciplinary Team. Mineralocorticoid antagonist treatment (eplerenone) was commenced to mitigate the risk of an intraprocedure hypertensive crisis because of a potential aldosterone surge.

## Outcome and Follow-up

The day after MWA, potassium supplements were discontinued, with serum levels maintained within the reference range. Moreover, serial measurements of the patient’s plasma aldosterone and renin levels have been persistently normal with most recent values (taken 6 years after thermal ablation): plasma aldosterone 190 pmol/L (6.85 ng/dL), PRA 2.3 nmol/L/h, and ARR 83 (Fig. 1). From a blood pressure perspective, he has been able to significantly deescalate his antihypertensive therapy regimen and remains only on amlodipine 10 mg daily with optimal control (average readings 125/80 mm Hg). His findings are consistent with complete biochemical and clinical cure from PA.

## Discussion

PA is the commonest form of secondary hypertension and is present in 5% to 15% of hypertensive adults [1]. In unilateral disease, adrenalectomy remains the treatment of choice owing to observational evidence highlighting its superiority to medical management with regard to important cardiovascular outcomes, such as an attenuated risk of atrial fibrillation [1]. Moreover, outcome data from the multicenter and international Primary Aldosteronism Surgical Outcome Study revealed that adrenalectomy for unilateral PA normalized blood pressure (without aid of antihypertensive medications) in 37% of 705 patients and achieved marked clinical success (in terms of improved blood pressure and/or less antihypertensive medications) in an additional 47%. Furthermore, complete biochemical success (correction of hypokalemia if it was present presurgery and normalization of ARR) was observed in 94% of 699 patients.

Minimal access adrenalectomy is considered the gold standard approach for small, nonmalignant adrenal tumors, with laparoscopic transperitoneal adrenalectomy being the first such approach [1]. Retroperitoneoscopic adrenalectomy is an alternative approach that appears to have some advantages over the laparoscopic approach in selected cases [2]. This involves the patient in the prone position; the gland is approached retroperitoneoscopically and so does not require breach of the peritoneal cavity or manipulation of intraperitoneal organs. Recent further meta-analysis of 775 patients (44% using the retroperitoneoscopic approach and 56% laparoscopic approach suggests that this technique presents the advantages of less estimated blood loss, earlier postoperative oral intake, and shorter hospital stays [3]. The role of a partial adrenalectomy, in which the adenoma and surrounding adrenal are removed, leaving part of the presumed normal adrenal gland, is an established alternative to a complete adrenalectomy in APA [4]. This is undertaken routinely or selectively as in the authors’ institution and does expose the patient to a small risk of persistence in the event of unilateral adrenal hyperplasia or multiple adenomas as in the case presented.

Beyond patients with PA resulting from bilateral disease, medical management has traditionally been reserved for those with a solitary adrenal adenoma who are unlikely to be cured by surgery, who are unfit for an operation, or who express a preference for medical management. However, as demonstrated in our patient, thermal ablation may be an effective and safe alternative. Indeed, this approach has received considerable attention as a minimally invasive technique for the treatment and palliation of various primary and secondary solid tumors, including hepatic, lung, renal, and breast neoplasms. In brief, percutaneous thermal ablation involves the use of thermal energy delivered directly to a tumor to induce necrosis, with radiofrequency ablation (RFA; using an electric current) and MWA (using an electromagnetic field) the most common modalities [5]. Hence, given the oncological effectiveness of thermal ablation, its application has been extrapolated to functional benign adrenal lesions, with the first reported case of a patient with unilateral PA resulting from an APA treated using RFA only described in 2004. Beyond the setting of adrenal malignancies (adrenocortical carcinoma or adrenal metastases), MWA is significantly less reported in the literature, whereas numerous publications have reported on the clinical outcomes of RFA as a primary procedure in PA [6-9]. To our knowledge, this is the first published case of thermal ablation as a salvage procedure following unsuccessful adrenal surgery.

Of note to our patient's management, the use of MWA is particularly novel given that previous case series reporting on the use of thermal ablation to treat unilateral PA have used RFA [6-9]. This is significant given that based on other tumors, MWA offers numerous advantages over RFA. For instance, MWA is more effective in destroying neoplastic cells by producing larger and more uniform ablation zones compared with RFA and is less likely to leave residual tumor cells that would recur over time [5]. Moreover, MWA is not susceptible to the heat sink effect (ie, the dissipation of heat via blood vessel perfusion) [5]. It is also significantly faster (at least 3 times) than RFA in creating the same ablation volume, shortening the overall procedural time under general anaesthetic [5]. The major complications include vascular, mechanical (ie, collateral damage to surrounding tissues), and infectious (ie, of surrounding tissue and ablation zone) factors. Notably, based on evidence from liver metastases, complication rates following thermal ablation are observed to be low, with MWA resulting in lower major complication rates than RFA [10].

To date, 3 main retrospective studies have been undertaken comparing long-term outcomes in patients with unilateral PA resulting from an APA undergoing either laparoscopic adrenalectomy or CT-guided percutaneous thermal ablation (specifically RFA). In the first publication from 2016 reporting on 44 patients (12 in the RFA group and 32 in the LA group), both interventions resulted in normokalemia in all patients [6]. Regarding cure from hypertension, this occurred more frequently in the adrenalectomy group (38% vs 17%), although this difference was not statistically significant [6]. In a separate and larger study from 2016 of 63 patients (36 in the RFA group and 27 in the LA group), resolution of PA was seen in 92% of the patients treated with RFA and all the patients treated by LA [7]. Furthermore, whereas hypokalemia resolved in all patients in both groups, hypertension resolved more frequently after LA compared with RFA (70% vs 36%) [7]. Finally, in a more recent study of 34 patients (10 in the RFA group and 24 in the LA group), more patients had hypertension cured and blood pressure controlled following LA (29% vs 0% and 96% vs 50%, respectively) [8]. Turning to perioperative outcomes and safety, a meta-analysis of 4 retrospective studies (including 2 of the publications [6, 7] described previously) demonstrated that RFA was associated with significantly shorter operative time, less operative blood loss, and shorter hospital stay compared with LA [9]. Collectively, this series of studies provides early promise of efficacy, suggesting that thermal ablation may achieve clinical outcomes that approach LA for patients with unilateral PA resulting from APA. Notably, these studies are small, retrospective, and nonrandomized, meaning that at present there is insufficient evidence for valid comparison with surgery. It is timely that the first multicenter randomized and prospective study comparing RFA with laparoscopic adrenalectomy as an alternative treatment for unilateral PA is currently recruiting (ClinicalTrials.gov identifier NCT05405101). Another fruitful area for future study would be comparing short- and long-term outcomes for PA patient receiving RFA vs MWA.

To this end, we present a patient with unilateral PA who received MWA following unsuccessful adrenal surgery and is now cured from PA. We demonstrate that in selected patients (such as those who are unfit or reluctant for surgery), MWA may become an important new addition to the treatment armamentarium for the management of this prevalent endocrinopathy.

## Learning Points

- Adrenalectomy via a minimally invasive approach remains the treatment of choice for primary aldosteronism (PA) resulting from an aldosterone-producing adenoma (APA).
- A partial (vs complete) adrenalectomy may expose a patient to a small risk of persistent PA in the event of unilateral adrenal hyperplasia or multiple adenomas.
- Thermal ablation may be a justifiable alternative to adrenalectomy for selected patients, for example those who are unfit or reluctant for surgery.
- Beyond radiofrequency ablation (which is well-reported in the literature), microwave ablation may also result in good clinical outcomes for patients with PA resulting from APA.
- At present, there is insufficient evidence for valid comparison of thermal ablation with adrenalectomy with regard to resolution of PA, hypertension, and hypokalemia, with future multicenter randomized and prospective trials much warranted.

## Data Availability

Not applicable to this article because no datasets were generated or analyzed during the study.

## Abbreviations

APA: aldosterone-producing adenoma
ARR: aldosterone:renin ratio
CT: computed tomography
RFA: radiofrequency ablation
MWA: microwave ablation
PA: primary aldosteronism
PRA: plasma renin activity
RR: reference range
